# Label-free detection of nanoparticles using depth scanning correlation interferometric microscopy

**DOI:** 10.1038/s41598-019-45439-x

**Published:** 2019-06-21

**Authors:** Ugur Aygun, Hakan Urey, Ayca Yalcin Ozkumur

**Affiliations:** 1 Department of Electrical and Electronics Engineering, Koç University, Istanbul, Turkey; 2 Research Center for Translational Medicine (KUTTAM), Koç University, Istanbul, Turkey; 3 Department of Electrical and Electronics Engineering, Bahçeşehir University, Istanbul, Turkey; 4 Department of Electrical and Computer Engineering, Boston University, Boston, Massachusetts USA

**Keywords:** Imaging and sensing, Interference microscopy

## Abstract

Single particle level visualization of biological nanoparticles such as viruses and exosomes is challenging due to their small size and low dielectric contrast. Fluorescence based methods are highly preferred, however they require labelling which may perturb the functionality of the particle of interest. On the other hand, wide-field interferometric microscopy can be used to detect sub-diffraction limited nanoparticles without using any labels. Here we demonstrate that utilization of defocused images enhances the visibility of nanoparticles in interferometric microscopy and thus improves the detectable size limit. With the proposed method termed as Depth Scanning Correlation (DSC) Interferometric Microscopy, we experimentally demonstrate the detection of sub-35nm dielectric particles without using any labels. Furthermore, we demonstrate direct detection of single exosomes. This label-free and high throughput nanoparticle detection technique can be used to sense and characterize biological particles over a range between a few tens to a few hundred nanometers, where conventional methods are insufficient.

## Introduction

Direct detection and quantification of synthetic and naturally occurring nanoparticles is critical in many healthcare applications including determining viral load for diagnostics of infectious diseases^1^, assessment of quality of drug delivery reagents for therapy^2^, and discovery and evaluation of new biomarkers such as exosomes for disease diagnosis and treatment monitoring^3^. Clinically important nanoparticles have relatively low concentration, hence instead of ensemble detection, single particle level detection has been desired^4^. For visualization of nanoparticles, electron microscopy has been the gold standard. However, the complex instrumentation, low throughput, and the vacuum requirement that damages biological samples make its practical implementation in clinical settings inadequate. On the other hand, optical detection of nanoparticles is a challenging problem due to their small size and low dielectric index: Viruses typically range from 20 nm to a few hundred nanometers, whereas exosomes are 30–100 nm in size. Direct visualization based on elastic scattering-based techniques is therefore not straightforward, and a contrast mechanism is required. A popular contrast mechanism is fluorescence-based detection that most commonly provide ensemble measurements. Advanced microscopy techniques such as STORM^5,6^, Stimulated Depletion Emission (STED) Microscopy^7–9^, and Photoactivated Localization Microscopy (PALM)^10^ generate high resolution images of fluorophore tagged nano-structures^11,12^ with single particle resolution, however they require labelling of nanoparticles that may alter their properties and the outcome of the assay of interest^13,14^. Furthermore, these advanced imaging techniques are also low throughput methods, and especially for biosensing applications instead of generating high resolution images, simply detecting and counting the particles of interest with a large field of view is needed^15^.

To overcome the limitations of labelling, methods that employ label-free detection schemes including surface plasmon resonance (SPR) imaging microscopy^16^, photonic crystal enhanced microscopy^17^, lensless holographic microscopy^18,19^, dark field microscopy^20^ and interferometric microscopy have been successfully developed. The bottleneck in label-free optical detection of nanoparticles is the weak light-particle interaction for subwavelength-sized particles: the scattered field is very low for nanoparticles due to the size scaling factor (~1/*V*^2^, where *V* is the volume of the particle). Furthermore, biological nanoparticles have refractive indices that are close to the measurement medium, providing extremely low contrast for scattering based detection. Thus, scattered signal from nanoparticles can be easily overwhelmed by the noise factors associated with the detector, optical system, and sample substrate.

One way to modify the size scaling factor is to use interferometry, where scattering signal is combined with a reference signal^21,22^. One of the most successful implementation of this method is interferometric scattering (iSCAT) microscopy^23,24^, in which, detection of single virus^25^, single proteins^26,27^, and visualization of lipid membrane formation^28^ is demonstrated. iSCAT uses standard cover glass as a sample substrate and scattered light from the nanoparticles is interfered with a reference beam which is reflected by the interface of medium and the cover glass. Noise due to impurities in sample substrate and light source is eliminated by subtracting the background and frame averaging^26^ using high frame rate cameras with a trade-off in the field of view. Another interferometric detection method called Single Particle Interferometric Reflectance Imaging Sensor (SP-IRIS) uses a special sample surface, which is a layered substrate where the thickness of the layer is optimized for optimum interferometric signal^21,29^. SP-IRIS has been successfully utilized for the detection of viruses^30,31^ and exosomes^32^. In a recent work, by implementing aperture-shaping filters, detection of 50 nm polystyrene particles have been demonstrated^33^ in air.

Size detection limit in interferometric detection methods is determined mainly by the signal-to-noise ratio (SNR) of the system. In this paper, we utilize the defocusing response of dielectric nanoparticles to enhance the contrast of interference-based detection. We propose a method termed Depth Scanning Correlation (DSC) Interferometric Microscopy, where depth scan images of the immobilized nanoparticles on top of a sample substrate are acquired, and a correlation analysis is performed to enhance the visibility of the nanoparticles while diminishing the noise in the background. We showed that DSC enhances the contrast of interference-based detection and improves both the signal-to-noise ratio (SNR) and the detection limit.

## Results and Discussion

**Figure 1:**
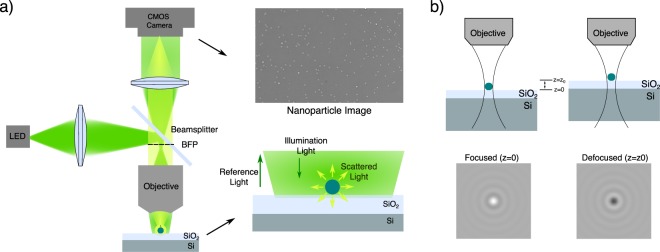
Experimental configuration of the interferometric imaging system. (**a**) Optical Setup. An LED is focused on near back focal plane of the microscope objective to have slightly converging illumination on sample substrate (See Methods). Scattered light from nanoparticles and the reflected reference light is collected and imaged to a camera plane. A layered Si/SiO_2_ with 100 nm oxide thickness is used as a sample substrate. (**b**) Defocusing Response. Upon defocusing, the path length between the reference and scattered light changes, hence constructive and destructive interference can be observed.

For visualization of the nanoparticles, we have constructed a wide-field interferometric microscope based on SP-IRIS^21^ (Fig. 1). Details of the optical setup is given in the Methods section. In brief, Koehler configuration is adapted for wide-field illumination, and a sample substrate of Si/SiO_2_ with 100 nm thick oxide is used. The scattered field from nanoparticles and the reference field reflected from the layered substrate is imaged onto a CMOS camera using the same 40x objective as used in illumination. The detected signal intensity can be written as

1 $${{\rm{I}}}_{det}={|{{\rm{E}}}_{{\rm{r}}{\rm{e}}{\rm{f}}}+{{\rm{E}}}_{{\rm{s}}{\rm{c}}{\rm{a}}}|}^{2}={{\rm{r}}}^{2}{{\rm{E}}}_{{\rm{i}}{\rm{n}}{\rm{c}}}+{{\rm{s}}}^{2}{{\rm{E}}}_{{\rm{i}}{\rm{n}}{\rm{c}}}+2.{\rm{r}}.\,{\rm{s}}.\cos ({\varphi }_{r}-{\varphi }_{s}).{{\rm{E}}}_{{\rm{i}}{\rm{n}}{\rm{c}}}$$

where *E*_*inc*_ is the incident field, *r* is the reflection coefficient of the layered substrate, *s (~1/V)* is the scattering amplitude of the nanoparticle and *ϕ*_*r*_ *−* *ϕ*_*s*_ is the phase difference between the reference and scattering fields. Note that in contrast to purely scattering based techniques, where particle signal is scaled with the square of the volume, the interference term in interferometric techniques (Eq. (1)) is scaled with *s* and thereby the volume of the particle.

The normalized signal on the detector can be written as,

2 $${I}_{n}=\frac{|{E}_{sca}+{E}_{ref}{|}^{2}}{{|{E}_{ref}|}^{2}}\approx \frac{2|{E}_{sca}|\cos ({\varphi }_{r}-{\varphi }_{s})}{|{E}_{ref}|}$$

Ignoring the DC term and assuming that |E_sca_|^2^ is small and can be ignored for nanoparticles, the total signal or the contrast of the nanoparticle image is mainly dominated by: (i) the amplitude of the scattered field |E_sca_| (envelope term i.e. Gaussian function) and (ii) the phase difference between the reference and scattered fields ($\cos ({\varphi }_{r}-{\varphi }_{s})$). Our implementation is a common path interferometric configuration in which nanoparticles are immobilized on top of a substrate. Therefore, the physical distance between nanoparticles and the sample substrate cannot be changed as in double path configurations such as Michelson interferometer. However, as we show in the simulations of dipole emission fields (Fig. S1), the scattered field on a layered substrate is mainly composed of higher angular components in contrast to the specularly reflected reference field. Any change in the axial position of the sample substrate (change in the objective-to-sample distance) introduces an optical path difference between the reference and scattered fields. This phase difference together with the intensity change of the scattered field modifies the detected signal.

In Fig. 1b, simulation results of the defocusing response of dielectric nanoparticles on top of layered substrate is given. In the simulations, nanoparticles are modelled as point dipoles with an orientation determined by the illumination field^34^, by following the physical model given in a recent work^35^. Due to spatial incoherence of the light source (LED), illumination field is modelled as incoherent sum of plane waves covering the illumination angle defined by NA of the objective. For each plane wave, the image of the dipole is calculated using the PSF of the imaging system. In the final step, the image of the particle is calculated by summing the individual dipole images. Nanoparticle image contrast in the final image is highly sensitive to the size of the particle (Fig. S2), as well as the axial location of the particle with respect to the substrate surface^29,36^.

In conventional SP-IRIS data acquisition and analysis pipeline, a defocus scan is acquired to identify a nominal focal plane, and images captured at that specific plane are analysed. Detection of exosomes^32^, and viruses^30,37^ have been demonstrated with this method in earlier work. However, due to the variability in the axial position of the immobilized nanoparticles both due to size of the particle or morphological variations in the surface capture probes, particle discrimination and size determination using the images captured at a single plane can be misleading. In order to improve the robustness of the visualization and quantification of nanoparticles, recently a “differential intensity image” concept is introduced^29^. In this technique, a z-stack is acquired by sweeping the axial position of the sample, and for each pixel, maximum and minimum intensity values are determined. The final difference image is composed of the difference in this peak to peak variations for each pixel. This approach increased the visibility of the nanoparticles and eliminated the inaccuracies due to variation in axial position for different particles. However, visibility (or SNR) can be further enhanced using the trend (all of the frames) in the z-stack instead of using only peaks (two frames) for application that require detection of smaller nanoparticles.

**Figure 2:**
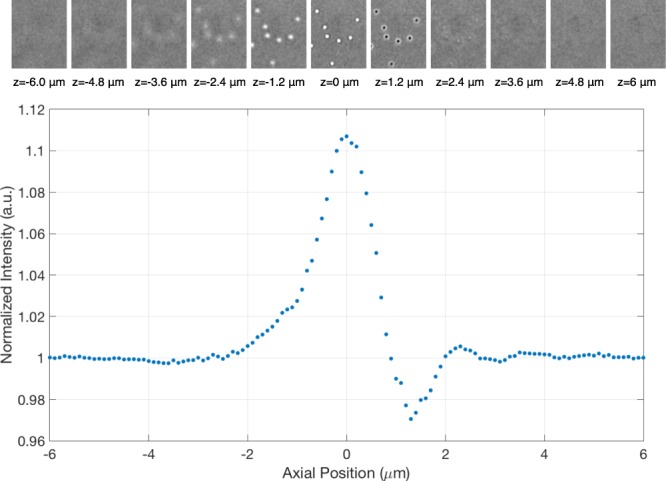
Experimental defocusing response. Polystrene nanoparticles with 100 nm in diameter are immobilized on top of sensor surface. By changing the axial position of the sample stage, images are captured, and the particle contrast value, which is the normalized intensity of a camera pixel as a function of axial position, is calculated. Figure illustrates the particle contrast for one pixel corresponding to the center of one of the particles in the above images.

To capture defocused particle images, the sample is placed on a piezo-stage and the axial (z) position is modulated with intervals of 100 nm. In Fig. 2 defocusing response of polystyrene (PS) nanoparticles with 100 nm diameter is shown. Similar to the simulations, the contrast of the nanoparticle image is highly sensitive to the axial position of the sample. Particles can display positive or negative contrast according to their axial positions.

**Figure 3:**
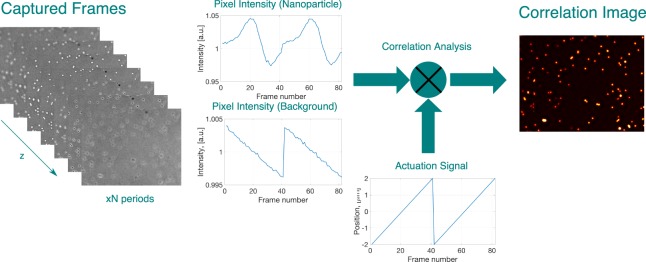
Depth Scanning Correlation Enhancement Procedure: An image stack is captured by actuating the sample stage in a saw tooth pattern and cross correlation analysis is performed between the pixel intensity value and the actuation signal (position of the sample stage) for each pixel individually. The correlation image is composed of calculated cross-correlation values.

In order to benefit from this defocusing behaviour and selectively enhance the nanoparticle response, we introduce the depth scanning correlation (DSC) technique as shown in Fig. 3. To discriminate particles from the background, the sample stage is actuated in z-direction and a cross-correlation analysis is performed between the actuation signal and every pixel in the captured images (Fig. 3). Pearson correlation coefficient *ρ* is calculated for every pixel location (x, y) as follows,

3 $${{\rm{I}}}_{{\rm{corr}}}({\rm{x}},{\rm{y}})={\rm{\rho }}({\rm{x}},{\rm{y}})=\frac{1}{{\rm{N}}-1}\sum _{{\rm{z}}={{\rm{z}}}_{0}}^{{{\rm{z}}}_{{\rm{N}}-1}}\,(\frac{{{\rm{I}}}_{{\rm{z}}}({\rm{x}},{\rm{y}})-\overline{{\rm{I}}({\rm{x}},{\rm{y}})}}{{{\rm{\sigma }}}_{{\rm{I}}}})(\frac{{\rm{R}}({\rm{z}})-\bar{{\rm{R}}}}{{{\rm{\sigma }}}_{{\rm{R}}}})$$

where *I*_*z*_*(x*, *y)* is the pixel’s intensity captured at axial position *z*, *R(z)* actuation signal’s level at z, *σ*_*I*_ and *σ*_*R*_ are the standard deviation of pixel intensity and actuation signal respectively; and $\,\overline{I(x,y)\,}$ and $\bar{R\,}$ mean values of pixel intensity and actuation signal over one period. The final correlation image is composed of the correlation values of each pixel. According to Eq. 3, *ρ* can get values between −1 and 1. *ρ* = *0* corresponds to highly uncorrelated signal, whereas *ρ* = *1* means highly correlated signal (Negative values correspond to anti-correlated behaviour). In order to obtain a highly correlated signal for the background signal, and thus distinguish nanoparticles from the background, the illumination is slightly tuned to have a converging beam on the sample so that the background will vary with z-scan (see Methods). In this way, an inverse relation between the background signal intensity and the axial position (actuation signal) of the sample is achieved as shown in Fig. 3, with a <0.5% variation in background signal over one period.

**Figure 4:**
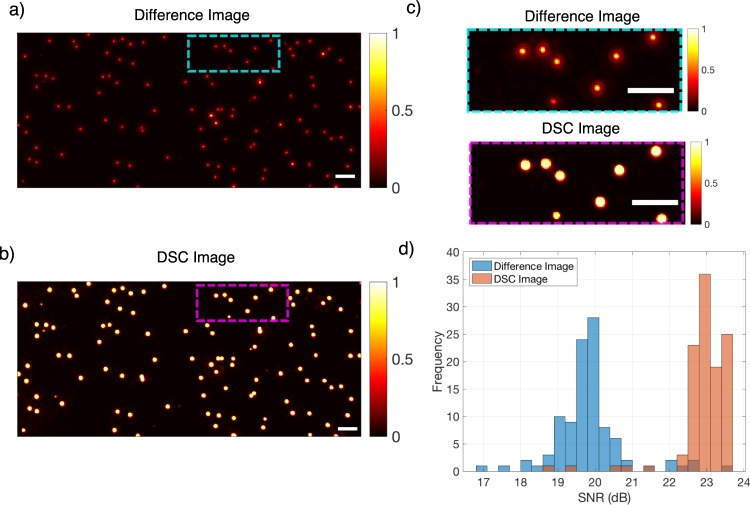
(**a**) Difference and (**b**) DSC image (5 µm defocus range) of 100 nm PS particles. (**c**) Zoomed image of Difference (top) and DSC image (bottom). (**d**) Detected particle SNR distribution for both methods. Scalebar is 5 µm.

The proposed technique is used to enhance the visibility of the nanoparticles. The performance of the method is first tested with polystyrene (PS) nanoparticles. Particles with a mean diameter of 100 nm and 50 nm are immobilized on separate substrates, and after an initial coarse focusing, the sample stage is actuated with 100 nm steps for a total of 10 µm z-displacement. Captured image stacks are processed to calculate (i) difference image (in which a peak to peak difference is calculated for each pixel, see Methods for details) and (ii) DSC image. In DSC image generation a simple search algorithm to find the optimum axial sweep region that maximizes the SNR of the particles is implemented. In Fig. 4, the difference image (a) and the DSC image (b) for 100 nm PS particles are shown. The enhancement of 2-fold in the signal to noise ratio (SNR) of individual nanoparticles with the DSC method is demonstrated (Fig. 4d).).

**Figure 5:**
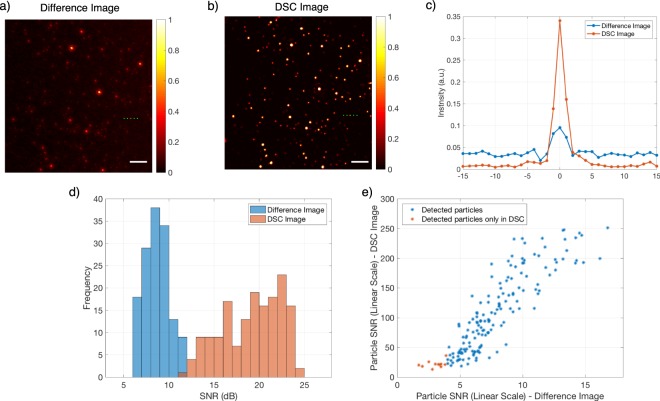
(**a**) Difference and (**b**) DSC image of the PS particles with the mean diameter of 50 nm (**c**) lineprofile corresponding to green line cut in (**a**) and (**b**). (**d**) SNR distribution of detected particles in both methods. (**e**) Particle SNR comparison for both methods. 2 µm defocus range is used in the analysis. Scalebar is 5 µm.

In Fig. 5, the difference and DSC images for PS nanoparticles with 50 nm mean diameter are given. Similar to the 100 nm particles, the SNR is significantly improved using the DSC technique. Note that for the PS particles of 50 nm average diameter, a heterogeneity in the SNR distribution is observed (Fig. 5d). The heterogeneity in particle size distribution is validated with SEM measurements for a direct comparison with the SNR measurements (Figs S3 and S4). In Fig. 5e, a comparison of the particle SNR values for both methods is given. According to Rose criterion (SNR > 4)^38^, some of the particles are only detected in DSC image. Furthermore, SNR range of detected particles are also increased in DSC analysis, which is critical for size discrimination. In contrast to label-based methods, particle signal in interferometric microscopy carries information about the size of the particle (intensity is scaled with the volume of the particle). Therefore, a direct relation between the SNR and the particle size can be generated.

**Figure 6:**
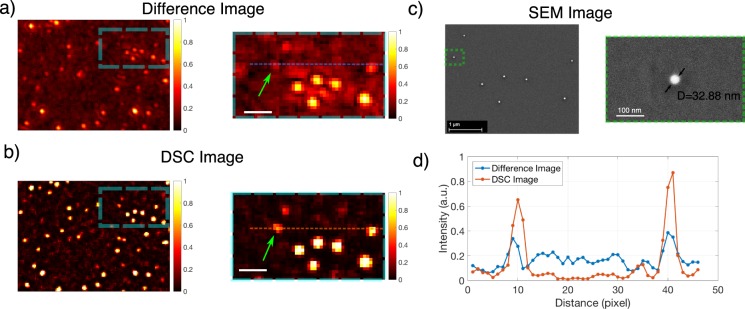
(**a**) Difference and (**b**) DSC (image of various sized polystyrene nanoparticles. Scalebar is 1 µm. Some of the particles visible in the DSC image are not detectable in the difference image (**c**) SEM image of the same region and zoomed in version. (**d**) Lineprofiles along the dashed lines shown in (**a**) and (**b**), for difference image and DSC image respectively.

One can argue that whether enhancing the contrast or SNR of the particles which are already detectable in an image is valuable or not. As long as there is a detectable particle in the image, its contrast can easily be improved by applying a threshold or subtracting the background. What is of more interest is to improve the detection limit (smallest detectable particle). In order to demonstrate that depth scanning correlation enhancement enables detection of smaller nanoparticles over the analysis from difference images alone, we performed experiments that incorporated smaller nanoparticles. In Fig. 6, we present a comparison between images obtained with both methods. In Fig. 6c, Scanning Electron Microscope (SEM) images of the same region are given. The diameter of the smallest particle (32.88 nm) is measured by focusing to the individual particle with SEM (Fig. 6c-right). This particle is detected in the DSC image with an SNR of 14.93 (11.74 dB) indicated with a green arrow (Fig. 6b-right)

**Figure 7:**
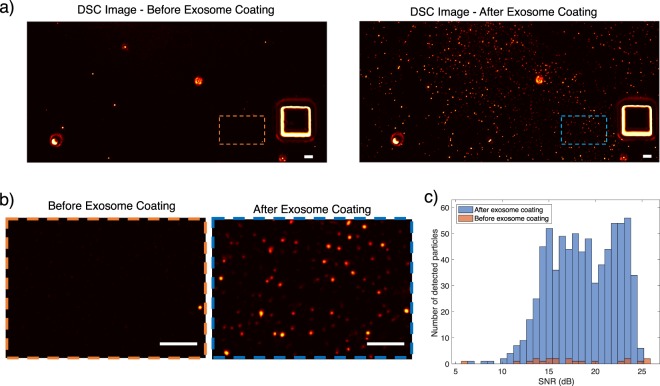
Exosome Detection: Unlabelled exosomes are immobilized on the sample substrate and visualized. (**a**) DSC image prior to exosome immobilization (left) and after exosome immobilization(right). (**b**) Zoomed in images of (**a**). (**c**) SNR distribution of detected exosome particles both prior and after exosome incubation. Scalebar is 5 µm.

In order to demonstrate the capability of the system for detecting biological nanoparticles, we have performed experiments with single unlabelled exosomes extracted via a size-based filtering^39^ (See Methods section). Exosomes are immobilized on the surface by spin coating and depth scan images are acquired before and after exosome immobilization. Correlation images are formed as shown in Fig. 7a and zoomed in version in Fig. 7b. SNR distribution of the detected particles for both prior and after exosome incubation is given in Fig. 7c. Note that due to heterogeneity in the exosome size distribution, detected particles have wide SNR distribution.

## Conclusions

In this paper, a new method - DSC interferometric microscopy – is proposed for the detection of dielectric nanoparticles. It is shown that the integration of mechanical actuation to interferometric imaging can be used to further enhance the visibility of the nanoparticles. The utilization of defocusing improves the detection of the presence of particles due to their unique defocusing response. Using correlation analysis, this response can be used to selectively amplify the particles in the image, while suppressing the background. Similar to other interferometric detection techniques, the SNR of a particle carries information about the size of the particle (scaled with the volume). We experimentally showed that this method can be used for direct detection of dielectric nanoparticles as small as 32 nm in diameter without using any optical or mechanical resonant behaviour. Furthermore, label-free detection of exosomes is demonstrated. We anticipate that the presented method can be used for a wide range of applications ranging from sample characterization to diagnostics, where label-free detection of individual biological nanoparticles is needed.

## Materials and Methods

### Optical imaging system

Our imaging platform (Fig. 1) is a widefield interferometric microscope with high magnification. As a sample substrate, layered Si/SiO_2_ is used. The sample substrate is uniformly illuminated by focusing a green LED (M530L3, Thorlabs Inc. NJ, USA) to the back focal plane of microscope objective (Plan Fluor 40x, 0.8 NA, Nikon Instruments, Amsterdam, Netherlands). After having uniform illumination on the sample substrate, focusing lens near the light source is slightly moved to have slightly converging beam on the substrate. In this way, a relation between the axial position of the sample and reflected field is achieved. The reflected field from the sample substrate together with the scattered field due to the sample (nanoparticles) is collected by the same objective and focused to a CMOS camera (Point Gray USB Grasshoper 3.0). Images with a field of view ~280 µm × 180 µm are captured. The position of the sample is controlled by a high precision piezo stage (Micronix, USA).

### Data acquisition and analysis

The stage and the camera are controlled by a custom written MATLAB script. For DSC analysis, after a coarse focusing is done manually, the piezo stage is actuated in the axial (z-) direction, between −5 µm to +5 µm with respect to nominal focus, with a 100 nm step size. At each step, the image of the sample is captured (at 3 ms exposure time, 40 frames averaged for 100 nm PS particles and exosomes (Figs 4 and 7), and 160 frames averaged for smaller particles (Figs 5 and 6). To enhance the visibility of the nanoparticles, a cross correlation analysis is performed between each pixel of the image stacks and a reference waveform obtained from the axial position of the sample. For each pixel, a Pearson correlation coefficient (ρ) is calculated by the “corr” built-in function of MATLAB. Optimum defocusing range is determined by running the DSC image generation algorithm for different defocusing ranges and maximizing the average SNR for detected particles (Fig. S7).

In order to generate the difference image, the same image stack captured for the DSC analysis is used. First, each frame in the z-stack is normalized by dividing to background intensity value. Then, for each pixel in the image, a maximum value and a minimum value of the intensity in defocused images are detected and the difference is calculated.

### Sample preparation

Si/SiO_2_ substrates with 100 nm oxide are purchased from Silicon Valley Microelectronics and cut into 1 cm × 1 cm squares. After standard cleaning (sonicating in acetone, rinse with methanol and deionized water), substrates are dried with nitrogen. Polystrene nanoparticles are purchased from Nanocs Inc, and exosome samples are supplied by BAMM Lab, Stanford University. Exosomes are isolated using EXOTIC exosome isolation chip^39^. Both exosomes and polystyrene nanoparticles are immobilized on the surface by spin coating.

### SEM imaging

Scanning Electron Microscopy images are captured using Zeiss Ultra Plus Field Emission Scanning Electron Microscope at Koç University Surface Science and Technology Center (KUYTAM). All of the SEM images are captured after optical measurements are finished. It is observed that upon SEM imaging, visualization of the nanoparticles is enhanced in interferometric microscopy due to the destructive nature of electron microscopy which can easily lead to false interpretation of the results. Therefore, only images captured before SEM measurements are used in the analysis.

## Supplementary information

Supplementary Information
