# Author Correction: Label-free detection of nanoparticles using depth scanning correlation interferometric microscopy

**DOI:** 10.1038/s41598-019-53131-3

**Published:** 2019-11-08

**Authors:** Ugur Aygun, Hakan Urey, Ayca Yalcin Ozkumur

**Affiliations:** 1 Department of Electrical and Electronics Engineering, Koç University, Istanbul, Turkey; 2 Research Center for Translational Medicine (KUTTAM), Koç University, Istanbul, Turkey; 3 Department of Electrical and Electronics Engineering, Bahçeşehir University, Istanbul, Turkey; 4 Department of Electrical and Computer Engineering, Boston University, Boston, Massachusetts USA

Correction to: *Scientific Reports* 10.1038/s41598-019-45439-x, published online 21 June 2019

The Acknowledgements section in this Article is incomplete.

“The authors would like to thank Dr. Baris Yagci (Koç University-KUYTAM) for SEM analysis, Prof. Utkan Demirci (BAMM Lab, Stanford University) for kindly providing exosome samples and Prof. M. Selim Ünlü (Boston University) for introduction to IRIS technology and helpful discussions. This work has been supported by TUBITAK Grants 113E643 and 115E260. The authors gratefully acknowledge use of the facilities of Koç University Research Center for Translational Medicine (KUTTAM)”.

should read:

“The authors would like to thank Dr. Baris Yagci (Koç University-KUYTAM) for SEM analysis, Prof. Utkan Demirci (BAMM Lab, Stanford University) for kindly providing exosome samples and Prof. M. Selim Ünlü (Boston University) for introduction to IRIS technology and helpful discussions. This work has been supported by TUBITAK Grants 113E643 and 115E260. Ayca Yalcin Ozkumur received partial funding from the European Union’s Horizon 2020 research and innovation programme under grant agreement No 766466. The authors gratefully acknowledge use of the facilities of Koç University Research Center for Translational Medicine (KUTTAM)”.

