# Supplementary material for: Label-free detection of nanoparticles using depth scanning correlation interferometric microscopy
Source: Sci Rep. 2019 Jun 21;9:9012. doi: 10.1038/s41598-019-45439-x (PMC6588623; doi:10.1038/s41598-019-45439-x)
Supplement: Supplementary file 1 — Supplementary Information [file 41598_2019_45439_MOESM1_ESM.pdf]

Supplementary Information for

Label-free detection of nanoparticles using depth scanning correlation interferometric microscopy

Authors: Ugur Aygun[1.2], Hakan Urey[1,2], Ayca Yalcin Ozkumur[3,4,*]

Affiliations:

[1] Department of Electrical and Electronics Engineering, Koç University, Istanbul, Turkey
[2] Research Center for Translational Medicine (KUTTAM), Koç University, Istanbul, Turkey
[3] Department of Electrical and Electronics Engineering, Bahçeşehir University, Istanbul, Turkey
[4] Department of Electrical and Computer Engineering, Boston University, Boston, Massachusetts, USA

Correspondence:
*Ayca Yalcin Ozkumur
E-mail: aozkumur@gmail.com

1. Dipole Far Field Radiation on a Layered Substrate

Nanoparticles can be modelled as point dipoles as shown in Figure S1a. Far field radiation of a point dipole on a substrate can be written as [1]

$$\vec{E}_{\infty} = \begin{bmatrix} E_\theta \\ E_\phi \end{bmatrix} = \frac{k^2}{4\pi^2\epsilon_0^2}\frac{e^{ikr}}{r}\begin{pmatrix} \Phi_2\cos\theta\cos\phi & \Phi_2\cos\theta\sin\phi & -\Phi_1\sin\theta \\ -\Phi_3\sin\phi & \Phi_3 cos\phi & 0 \end{pmatrix}\begin{pmatrix} \mu_x \\ \mu_y \\ \mu_z \end{pmatrix} \quad \text{(S1)}$$

where

$$\Phi_1 = [e^{-ikz_0\cos\theta} + r^p(\theta).e^{ikz_0\cos\theta}]$$

$$\Phi_2 = [e^{-ikz_0\cos\theta} - r^p(\theta).e^{ikz_0\cos\theta}] \quad \text{(S2)}$$

$$\Phi_3 = [e^{-ikz_0\cos\theta} + r^s(\theta).e^{ikz_0\cos\theta}]$$

k is the wavenumber, μ_x, μ_y and μ_z are the point dipole moment components in cartesian coordinates (Figure S1-a), z_0 is the axial position of the point dipole, r^s and r^p are the Fresnel reflection coefficients for the *s*- and *p*- polarized light respectively.

Emission patterns at back aperture of the objective for different oriented point dipoles are simulated in Figure S1-b. Note that for all of the cases radiation is directed to higher angles. By using high NA objectives these photons can be collected. On the other if widefield illumination is used (low NA illumination), the direction of the reflected field will be nearly parallel to the optical axis. Therefore, even though phase of the scattered field and the reference field cannot be adjusted independently, phase difference can be changed by defocusing due to different angular components of these signals.

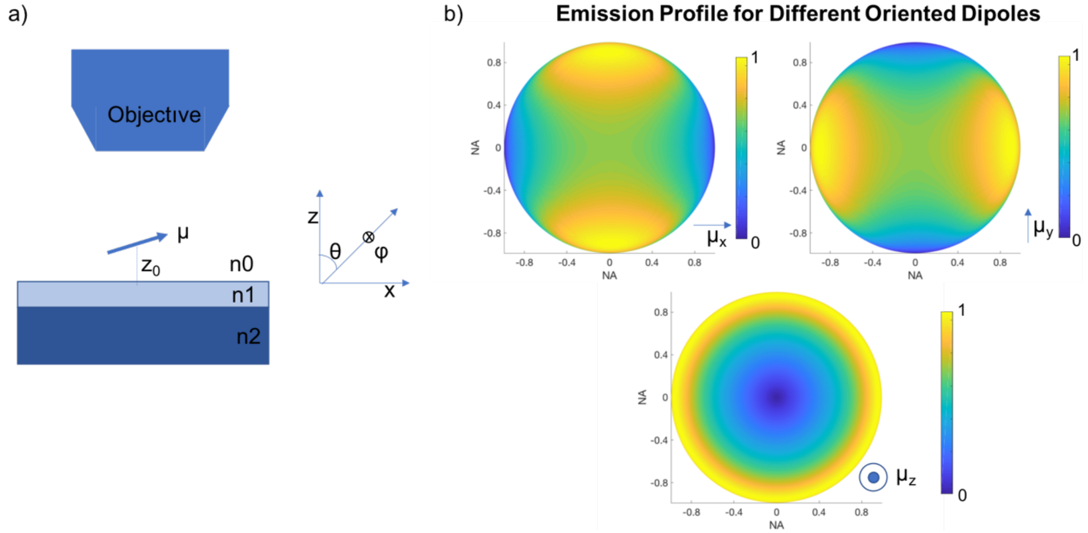

Figure S1: a) Coordinate system used for the calculations b) Calculated emission profiles for different oriented dipoles located on top of a layered substrate (n1=1, n2=1.4, n3=4, λ=525 nm).

2. Defocusing Response of Nanoparticles in Widefield Interferometric Microscopy

In widefield interferometric microscopy signal at detector plane due to a nanoparticle can be written as

$$I_t = |E_s + E_r|^2 = |E_s|^2 + |E_r|^2 + 2|E_r||E_s|\cos(\phi_s - \phi_r) \quad \text{(S3)}$$

Here E_s is the scattering field (scaled with the volume of the particle) and E_r is the reference field reflected from the substrate surface. Assuming $|E_s|^2$ is small for the nanoparticles, total signal is scaled with the volume of the particle. Furthermore, detected signal is highly sensitive to phase difference between the scattering and reference fields. By introducing defocus on imaging system (by moving sample with respective to objective) phase difference can be changed.

In general field due to a point dipole located at r_0 can be written as

$$E_s(r') = \overleftrightarrow{G}(r', r_0).\vec{\mu}$$

where G is the Green's dyadic (or PSF of the system) and μ is the point dipole vector. In the simulations we used the point spread function developed for widefield interferometric microscope in an earlier work[2].In Figure S2-a, experimental and simulated defocusing curves for polystyrene nanoparticles with 100 nm is plotted. In Figure S2-b simulated defocus curves for different sized particles are given. In Figure S2-c, expected signal to noise ratio for different sized particles is calculated for difference image method.

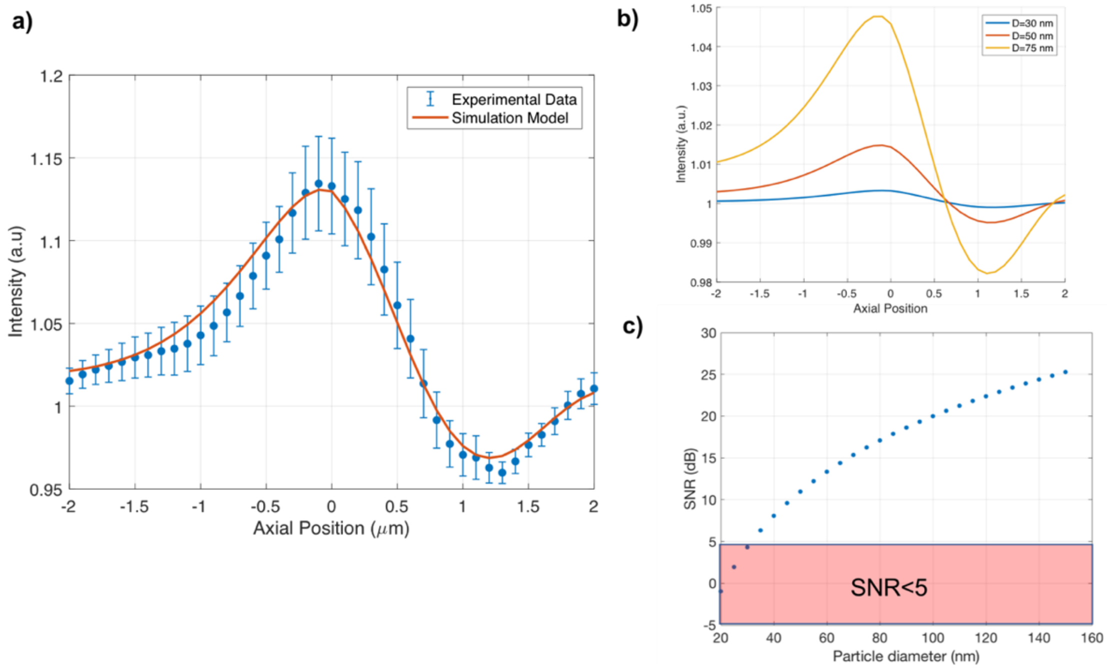

Figure S2: (a) Experimental and simulated defocus response for PS particles 100 nm in diameter (b) Simulated defocus responses for different sized PS particles (c) Simulated SNR values for different sized particles in widefield interferometric microscopy.

3. Verification with Scanning Electron Microscopy Images

In Figure S3-a Depth Scanning Correlation image of polystyrene particles with various size is shown. Zoomed in images and corresponding Scanning Electron Microscopy (SEM) images are given in Figure S3-b. Note that in DSC images contrast or SNR of the particles carries information about the size of the particle. On the other hand, aggregates or particles that are closer together than the diffraction limited resolution also appears as bright spots (*some particles in R3 and R4 region*). The diameter of the particles is determined by SEM images. Corresponding SNR values calculated in DSC images are plotted in Figure S3-c. Even though the number of measured particles is limited, there is a positive correlation between size and SNR.

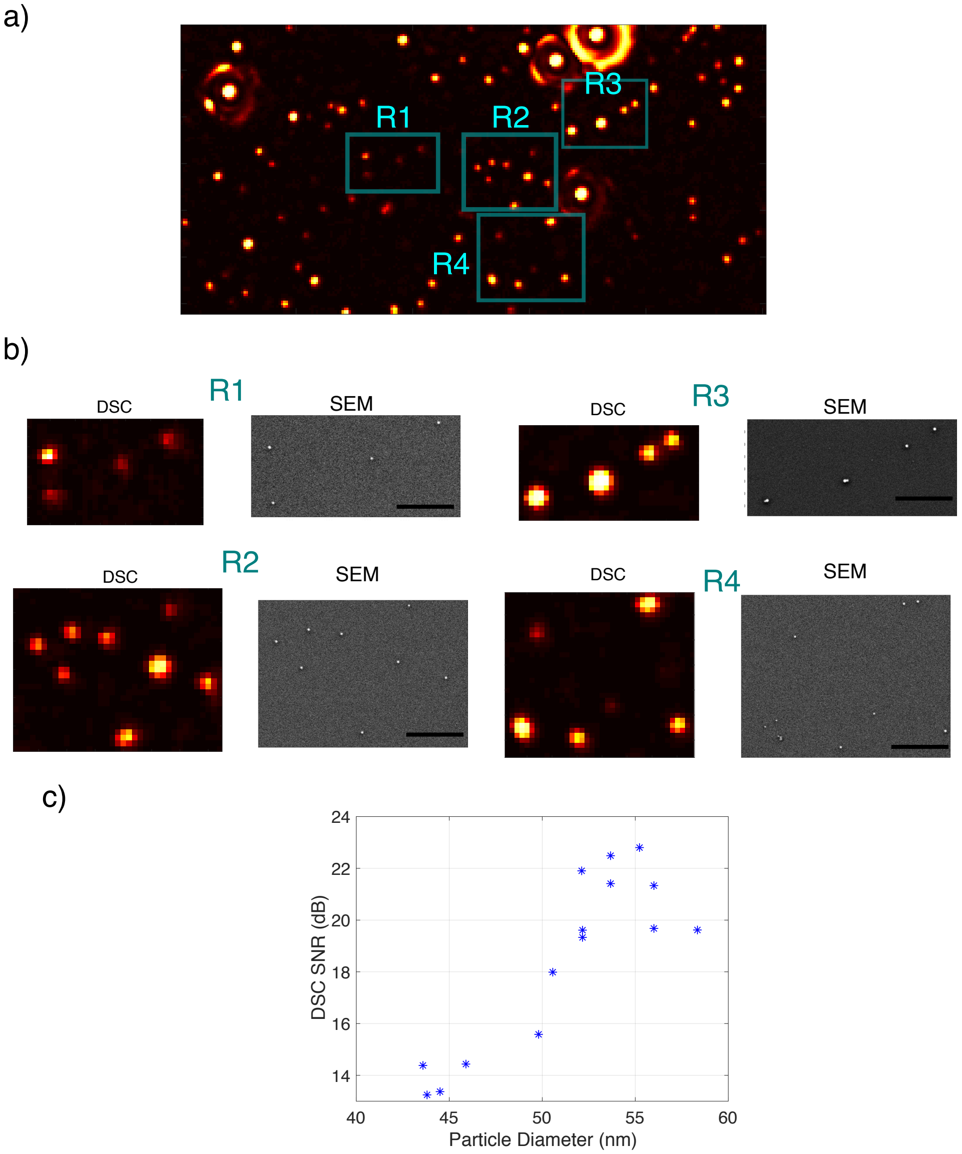

Figure S3: (a) DSC image of PS nanoparticles captured by using 2 µm defocus range (b) SEM images of corresponding regions (c) Variation of the measured SNR values with respect to the particle diameters measured with SEM for 2 µm defocus range. Scalebar is 1 µm.

In DSC technique defocus range used in the analysis affects the SNR of the measurements. Implementation of large defocus range is favorable in terms of decreasing the measurement noise, however for smaller particles change in interferometric signal due to defocusing is limited as can be seen in simulations given in Fig S2-b. Therefore, for smaller particles there is an optimum defocus range for maximum SNR (Figure S7-c). Measured SNR distribution for 1 µm defocus range is given in Figure S4. Although the dynamic range of the signal is lower (therefore size discrimination capacity) comparing to 2 µm defocus range case, detection of smaller particles is achieved.

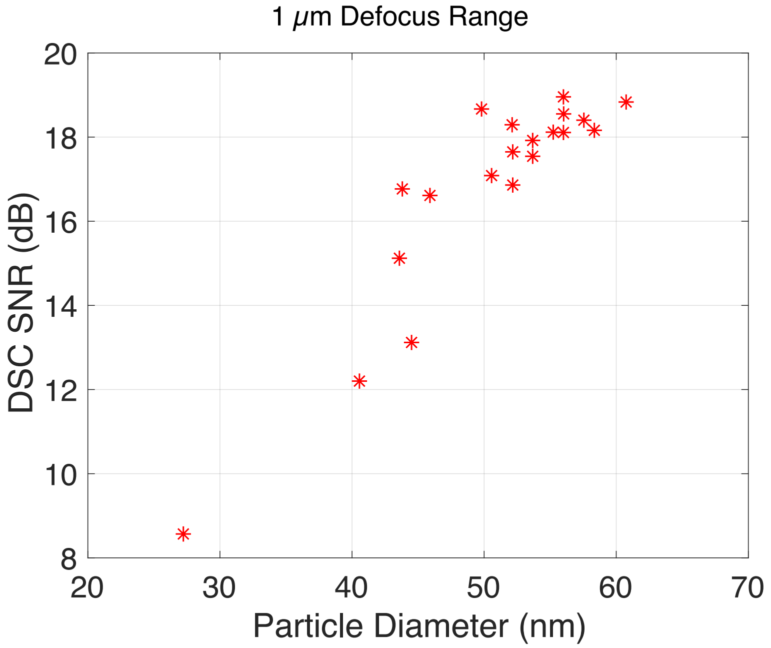

Figure S4: Variation of the measured SNR values with respect to the particle diameters measured with SEM for 1 µm defocus range.

4. Signal to Noise Ratio vs Defocus Range in Depth Scanning Correlation Interferometric Microscopy

Depth Scanning Correlation (DSC) technique utilizes the defocused images to enhance the visibility of the nanoparticles in widefield interferometric microscopy. Selection of optimum defocus range is critical to maximize the SNR of detected particles. A typical defocus response of two different particles is plotted in Figure S5.

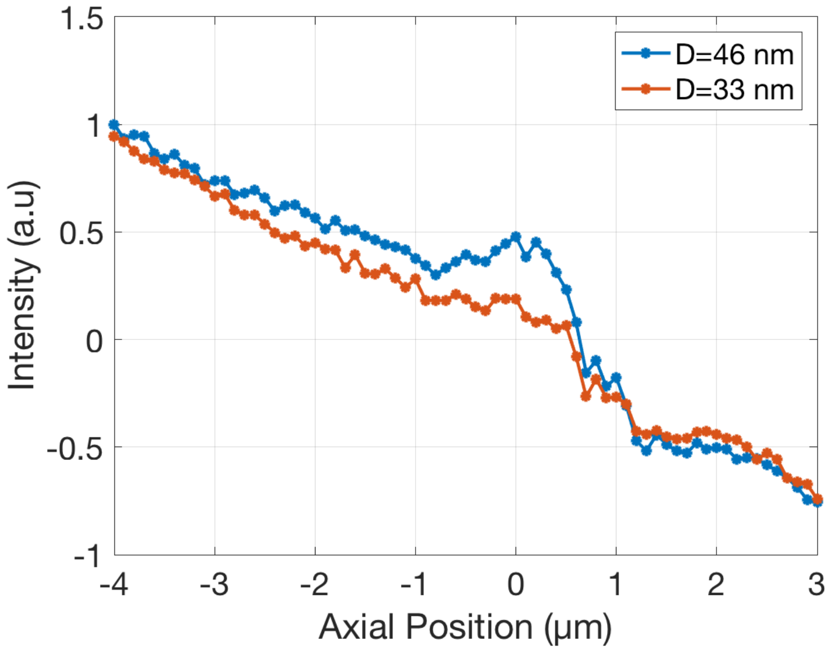

Figure S5: Experimental defocus response for PS particles with 46 nm in diameter (blue) and 33 nm in diameter(red).

In order to find optimum defocusing range for the DSC image generation, Pearson coefficients (Eq.2) for different defocus windows are calculated. Particle signals ($I_{corr,particle}$- $I_{corr,background}$) is shown in Figure S6 for these two particles. Particle signal is calculated as the average background intensity is subtracted from the average particle intensity. According to Figure S6, in order to have maximum signal total defocus length should be around 1.5 µm and the last frame utilized in the analysis should be captured at nominal focal plane (z=0).

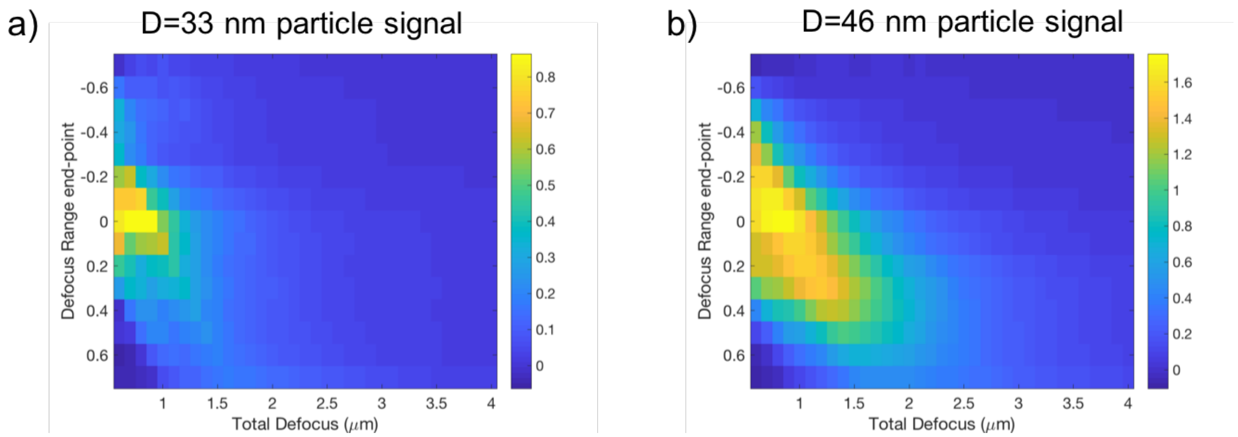

Figure S6: Calculated particle signals for different defocus windows

In Figure S7-a particle signal and background noise dependence to total defocus length is plotted (assuming the last frame is captured at z=0). Following a short increase, total signal is decreased by increasing the total defocus range. However, background noise which is the standard

deviation in background signal is also decreased by increasing the total defocus length. In Fig S6-b measured background noise is plotted. In Fig S7-c SNR for the particles is shown. One interesting result is the optimum defocusing range is a function of particle size. For this specific example in order to obtain highest SNR for D=33 nm particle z=[-0.9 µm, 0 µm], and for D=46 nm particle z=[-1.5 µm, 0 µm] should be used in the analysis.

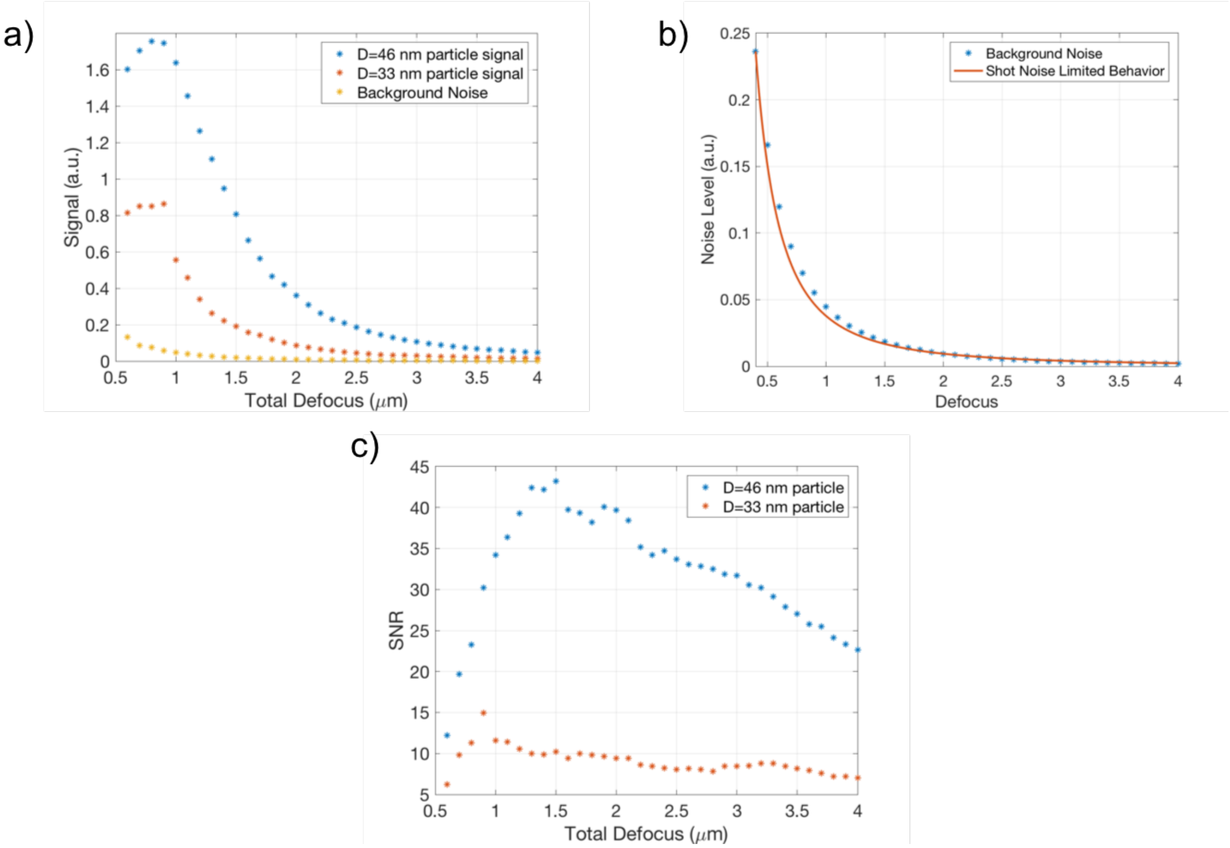

Figure S7 (a) Particle signal and (b) Background noise and (c) SNR dependence to total defocus range.

References

1. Novotny, Lukas; Hecht, B. *Principles of Nano-optics*. (Cambridge University Press, 2006).
2. Avci, O., Adato, R., Ozkumur, A. Y. & Ünlü, M. S. Physical modeling of interference enhanced imaging and characterization of single nanoparticles. *Opt. Express* **24,** 6094 (2016).
